# Prevalence of anxiety, depression, mania, insomnia, stress, suicidal ideation, psychotic experiences, & loneliness in UK university students

**DOI:** 10.1038/s41597-023-02520-5

**Published:** 2023-09-13

**Authors:** Umair Akram, Kamila Irvine, Maria Gardani, Sarah Allen, Asha Akram, Jodie C. Stevenson

**Affiliations:** 1 School of Psychology, University of Lincoln, Lincoln, UK; 2 Nuffield Department of Clinical Neurosciences, University of Oxford, Oxford, UK; 3 School of Health in Social Science, University of Edinburgh, Edinburgh, UK; 4 Department of Psychology, Northumbria University, Newcastle upon Tyne, UK; 5 Department of Psychology, The University of Sheffield, Sheffield, UK

**Keywords:** Human behaviour, Risk factors

## Abstract

Despite existing wellbeing services, university students remain particularly vulnerable to mental health difficulties. Therefore, this study was designed to provide a comprehensive assessment of the prevalence of psychiatric symptoms by using well validated scales with robust psychometric properties. More specifically, the current data provides crucial information concerning the prevalence of anxiety, depression, mania, insomnia, stress, suicidal ideation, psychotic experiences and loneliness amongst a sample of N = 1408 UK university students. A cross-sectional online questionnaire-based study was implemented. Online recruitment for this dataset began on September 17^th^, 2018, and ended on the 30^th^ July 2019. Eight validated measures were used: Generalized Anxiety Disorder Scale; Patient Health Questionnaire; The Mood Disorder Questionnaire; The Sleep Condition Indicator; The Perceived Stress Scale; Suicidal Behaviours Questionnaire-Revised; The Prodromal Questionnaire 16 (PQ-16); and the University of California Loneliness Scale. The dataset is available to other researchers and is provided on figshare. Information concerning the data records, usage notes, code availability and technical validation are presented. Finally, we present demographic information concerning psychiatric symptom prevalence.

## Background & Summary

Vulnerability to psychiatric distress and accentuation, or initial emergence of mental health difficulties remains particularly high amongst individuals persuing university level study^1,2^. The transition to higher education level study entails notable change to academic and psychosocial functioning. In particular, reduced parental support and oversight may increase independence and pressure to participate in social obligations, both occurring alongside increased academic demands^2^. In the UK, data concerning psychiatric wellbeing in this population yields a steady pattern of yearly deterioration. Here, over a ten-year period, the amount of students disclosing their difficulties to institutional wellbeing services evidenced a fivefold increase^3,4^. The most prominently reported difficulties amongst university students in this country include the symptom experience of insomnia, stress, anxiety, depression, suicidal ideation, substance abuse, and loneliness^5–13^. As a partial result of increased demand, a recent audit found many institutional student wellbeing services fail to meet the demand for support^14,15^. Crucially, several reports evidence institutional services to be arduous and often ineffective, generally remaining limited to general non-specific talking treatments, often lacking in a transdiagnostic or symptom-oriented cognitive behavioral therapeutic approach^16–18^.

Evidence concerning the nature of psychiatric difficulties in UK university students remains limited, and numerous existing studies disproportionately target specific symptoms and/or student sub-samples, typically comprised of individuals pursuing dentistry, pharmacy or medicine^19–31^. Whilst typical with large scale data collection, other studies frequently employed scales comprised of a single-item, or bespoke in-house, which often fail to capture symptom severity and specificity^32^. Therefore, this study was designed to provide a comprehensive assessment of the prevalence of psychiatric symptoms by using well validated scales with robust psychometric properties. More specifically, the current data provides crucial information concerning the prevalence of anxiety, depression, mania, insomnia, stress, suicidal ideation, psychotic experiences and loneliness amongst UK university students.

## Methods

### Design, sample and procedure

Adhering to the British Psychological Society’s Criteria of Human Research Ethics, the study was approved by Sheffield Hallam University’s Research Ethics Committee (Protocol number: ER7368595). Informed consent was provided by each student prior to the start of data collection. Here, participants confirmed that they have i) carefully understood the participant information; and ii) consent to participating in the study under the conditions described within the participant information. The information included the eligibility criteria: a current university student over the age of eighteen; the nature of the study protocol – completing a series of published questionnaires designed to assess various symptoms relating to mental health problems; the perhaps sensitive nature of some questions and statement noting that participants may terminate the study at any point; information concerning confidentiality; data storage and future use of anonymised data for publication online, including the data file; details as to how ethical approval was sought and the approval code; contact details of the primary researcher; and a supplementary GDPR summary statement which was required at the time (to facilitate the transition).

A cross-sectional online questionnaire-based study was implemented comprising of questions designed to determine the dimensional experiences of psychiatric symptoms which included: anxiety, depression, mania, insomnia, perceived stress, suicidal thoughts and behaviours, psychotic-like experiences, and loneliness. Demographic information concerning participant age, sex, ethnicity, course format (i.e., undergraduate or postgraduate) were also collected. Students from five UK universities (University of Durham, University of Glasgow, Northumbria University, Sheffield Hallam University, University of Sheffield) were recruited through institutional course research credit schemes, school social media pages, and faculty emails. Some courses in the UK which involve data collection as part of the student’s final year project provide the option for students to gain research credits in their first two years of study. Here, students may complete a research study and subsequently used the credits when collecting their own data in the final year. Emails to the head of head of each school was contacted to request permission to share an email advertising the study, and where applicable possibly to provide the aforementioned credits. The social media pages included any of the institutions schools that agreed to share a short twitter or Facebook post to followers.

Online recruitment for this dataset began on September 17^th^, 2018, and ended on the 30^th^ July 2019. Due to the ethical right to withdraw at any point, only complete responses were retained for analysis. Possible duplicate responses were identified where based on duplicate IP addresses, where none were found. This resulted in a sample of N = 1841 individuals clicked a hyperlink to the survey landing page, which was delivered using the Qualtrics platform (Qualtrics, Provo, UT), and 1408 respondents (mean age = 20.94 ± 4.42, range 18–56, 83% female) providing complete data (final response rate = 76.5%) for analysis. This sample size was appropriate for a 95% confidence level, surpassing the initial minimum target of N = 500 responses with an acceptable 4.5% margin of error^33^. Where course credit was sought, students were remunerated on completion. See Table 1 for participant demographic information.

### Measures

#### Anxiety

The 7-item Generalized Anxiety Disorder Scale (GAD-7) was used to examine symptoms of anxiety. Participants indicated how often in the last 2 weeks, they have been bothered by the seven core symptoms of generalized anxiety disorder^34^. Response choices for each item are: 0 = “not at all”; 1 = “several days”; 2 = “more than half the days”; and 3 = “nearly every day”. Total scores range between 0 and 21 with higher scores indicating higher levels of symptom severity. Alternatively, cut offs of ≥5, ≥10, and ≥15 can be used to identify mild, moderate, and severe anxiety levels, respectively. A score of ≤4 suggests minimal levels of anxiety symptoms. The GAD-7 has previously indicated good reliability, as well as criterion, construct, factorial, and procedural validity^34,35^. The internal consistency (Cronbach’s alpha) in the current study was *α* = 0.923.

#### Depression

The 9-item Patient Health Questionnaire (PHQ-9) examined the prevalence of depressive symptoms^36^. Each item corresponds to the nine key symptoms of depression as described by the DSM-5 criteria^37^. For each item, participants indicated the extent to which over the last two weeks they were bothered by each symptom on a scale of: 0 = “not at all”, 1 = “several days”, 2 = “more than half of the days” or 3 = “nearly every day”. The summation of each item yields a total score of depression severity ranging between 0 – 27 where increased levels of depression are reflected by higher total scores Alternatively, categorical cut offs of ≥5, ≥10, ≥15 and ≥20 can be used to identify mild, moderate, moderately severe, and severe depression levels, respectively. A score of ≤4 suggests minimal levels of depressive symptoms. The scale has been shown to demonstrate good criterion and construct validity^36^. The internal consistency of the scale in the present study was *α* = 0.903.

#### Mania

The Mood Disorder Questionnaire (MDQ) was used to examine symptoms of mania (range 0–13)^38^. Participants provided a response of yes/no when presented with a series of prompting questions. For example, “Has there ever been a time when you were not your usual self and…”. The summation of each item yields a total MDQ score where higher scores indicate increased levels of manic symptoms. The internal consistency of the scale in the present study was *α* = 0.845. To screen positive for possible bipolar disorder, all three parts of the following criteria should be met based on the initial 13 items and two additional questions. More specifically, participants must answer yes to ≥7 of the thirteen items; indicate cooccurrence of ≥1 items within the same period; and report moderate to serious consequences of the possible episode (i.e., inability to work; family, monetary or legal difficulties; aggressive behaviour).

#### Insomnia

Based on the DSM-5 criteria for Insomnia Disorder, the eight-item Sleep Condition Indicator (SCI) examined students insomnia symptomology during the last month the extent of insomnia symptoms^39^. Scored between 0–4, items determine perceived sleep onset latency, night-time awakenings, quality of sleep obtained, impairment of daytime functioning and, symptom frequency. The summation of items yields a total score between 0–32. Here, increased severity of insomnia symptoms are reflected by lower scores. For categorical use, the SCI can reliably (89%) determine probable insomnia^39^. Previous large-scale studies show an excellent degree of reliability (*α* = 0.89) and concurrent validity of the SCI^39^. Likewise, the current assessment of internal consistency yielded a value of *α* = 0.872.

#### Stress

The extent of perceived stress experienced by students over the previous four weeks was determined using the Perceived Stress Scale (PSS). Fourteen items, scored on a 5-point Likert type scale (0–4), are summed to provide total scores ranging between 0 and 40. Higher scores indicate higher levels of perceived stress^40^. Alternatively, cut offs of ≤13, ≥14, and ≥27 can be used to identify low, moderate, and high levels, respectively. The internal consistency of the scale in the present study was α = 0.866.

#### Suicidal ideation

Suicidal thoughts and behaviours was examined using the four-item Suicidal Behaviours Questionnaire-Revised (SBQ-R)^41^. In particular, the SBQ-R measures: lifetime ideation/attempt; frequency of ideation within twelve months; expressing suicidal ideation to another person, and probability of attempting suicide in the future. Responses for each question can be analysed individually and/or summated to produce a total score between 3–18 with higher scores indicating increased risk. For categorical use, A score of ≥7 indicates significant risk for suicidal behaviour (Sensitivity, 93% and Specificity 91%)^41^. The SBQ-R reliably yields an acceptable level of predictive and concurrent validity^42–44^. Indeed, of nineteen systematically reviewed psychometric tools designed to examine risk of suicidal ideation, the SBQ-R remained one of three recommended brief assessments of suicidal thoughts and behaviours^45^. The internal of the scale in the present study was *α* = 0.841.

#### Psychotic experiences

Life-time symptoms of psychotic experiences were measured using the Prodromal Questionnaire 16 (PQ-16)^46^. More specifically, participants provided a response of yes/no when presented with sixteen items related to the experience of positive, negative, and avolitic symptoms. The summation of yes responses generates a total score between (0–16). Higher scores reflect an increased number of psychotic symptoms. In adults, a score of ≥6 predicts diagnosis of psychosis with high sensitivity (87%) and specificity (87%)^46,47^. The internal consistency of the scale in the current study was *α* = 0.822.

#### Loneliness

Loneliness was examined using the twenty-item third version of the University of California Loneliness Scale (UCLA3)^48,49^. Here, participants rated a series of statements related to loneliness (e.g., “I feel isolated from others”, “I feel completely alone”) on a four-point scale (1 = never, 4 = often). After reverse scoring required items, the summation of each item response provides a total loneliness score ranging between 20–80. Higher scores indicate a greater degree of loneliness. It is possible to create groups based on the severity of loneliness following Cacioppo’s approach^50,51^, by selecting the upper (lonely group: total score ≥52–80), middle (middle loneliness group: total score ≥40–51) or lower (non-lonely group: total score ≤39) percentile of the distribution. The internal consistency of the scale in the present study was *α* = 0.936.

**Table 1:** Sample characteristics.

	N/Mean ± SD	% of Total Sample
**Age** (**Mean** ± **SD**)	20.94 ± 4.42	
**Sex** (**Assigned at Birth**)		
Male	238	16.8
Female	1169	83
Intersex	1	0.1
Prefer not to report	1	0.1
**Gender**		
Male	236	16.8
Female	1154	82
Non-binary	5	0.4
Transgender	1	0.1
Gender-nonconforming	3	0.2
Unsure	5	0.4
Prefer not to report	1	0.1
Not Listed/Other	2	0.1
**Ethnic origin**		
White - United Kingdom	1115	79.2
White - Irish	18	1.3
White – Other	80	5.7
White and Black Caribbean	11	0.8
White and Black African	5	0.4
White and Asian	16	1.1
Mixed/Multiple Ethnic Other	15	1.1
Indian	39	2.8
Pakistani	22	1.6
Bangladeshi	8	0.6
Chinese	35	2.5
Asian Other	12	0.9
African	11	0.8
Caribbean	4	0.3
Black African/ Caribbean Other	1	0.1
Arab	7	0.5
Other	9	0.6
**Course level**		
Undergraduate	1213	86.2
Postgraduate taught^*^	104	7.4
Postgraduate research^*^	23	1.6
Doctoral student	40	2.8
Postgraduate Other	28	2.0
**Institution**		
Sheffield Hallam University	499	35.8
University of Sheffield	215	15.3
Northumbria University	402	28.6
University of Durham	130	9.2
University of Glasgow	29	2.1
Other	130	8.4
Missing Data	15	1.1

**Note:** ±, Standard Deviation; *Postgraduate taught courses (i.e., MSc) involve a greater number of credit modules where students attended a taught session, typically a lecture and seminar, relative to those with a greater research and methodological focus. Both involve a research project, and statistical methods modules. Postgraduate research courses (i.e., MRes) may involve more hands on non-assessed modules where students interact with methodological research tools.

## Data Records

The dataset (demographics, psychiatric measures) has been anonymised and both individual datapoints for each psychometric measure and the scored data are available in CSV and SAV formats on Figshare [10.6084/m9.figshare.24052236.v2]^52^ and as part of the current supplemental information (see Appendix A). For reasons pertaining to copyright, the original PDF files including scoring information for each measure are not included in the appendix. However, the key scoring information is provided in the measures section, and original measures can be individually examined where necessary. In the current dataset, reverse coding was implemented where required. Finally, the total/mean or composite score for each psychiatric symptom was calculated and included to facilitate the usability of the dataset. Descriptions of calculated scores are provided in Table 2. Details of the data cleaning procedure are available in the following section.

**Table 2:** Variable codes for dataset.

Data Codes	
**Demographic Variables**	
Age	Participant age
Sex	Biological sex assigned at birth
Gender	Gender identity
Ethnicity – Ethnicity_6	Participant ethnicity
Course_Type	Participants current level of study
Institution	Institution student attended when providing the current data.
Institution_Other	Name of institution
**Individual Psychometric Items**	
GAD7_1 – GAD7_2	The seven individual items of the Generalized Anxiety Disorder 7^X^.
PHQ_1 – PHQ_9	The nine individual items of the Patient Health Questionnaire
MDQ_1 – MDQ_13	The thirteen individual items of the Mood Disorder questionnaire
MDQ_Frequency	Possible cooccurrence of items within the same period.
MDQ_Severity	Consequence severity of the possible episode.
SCI1 – SCI8	The eight individual items of the Sleep Condition Indicator
PSS_1 – PSS_10	The ten individual items of the Perceived Stress Scale^X^.
SBQ1 – SBQ2	The four individual items of the Suicidal Behaviours Questionnaire-Revised^X^.
PRO16_1 – PRO16_16	The sixteen individual items of the Prodromal 16^X^.
UCLA3_1 – UCLA3_20	The twenty individual items of the University of California Loneliness Scale UCLA3^X^.
**Psychiatric Symptoms Scored**	
GAD7_Anxiety	Summation of anxiety items
PHQ9_Depression	Summation of depressive items
MDQ_Mania	Summation of mania items
SCI_Insomnia	Summation of insomnia items
SBQ_Suicidal_Ideation	Summation of suicidal ideation items
P16_Psychotic_Exp_Sum	Summation of items assessing psychotic like experiences
UCLA3_Loneliness	Summation of items assessing loneliness

## Usage Notes

Data have been deidentified and are presented in the same manner across data files, which can be imported into most compatible statistical software packages. As noted, the dataset includes all individual datapoints for each de-identifiable psychometric measure. For each validated questionnaire, the initial authors scoring instructions were followed to calculate the relevant total, composite, and subscale variables which may be of interest. Data indicating the institution students were attending at the time may be used to split the data into cohorts.

## Technical Validation

**Fig. 1:**
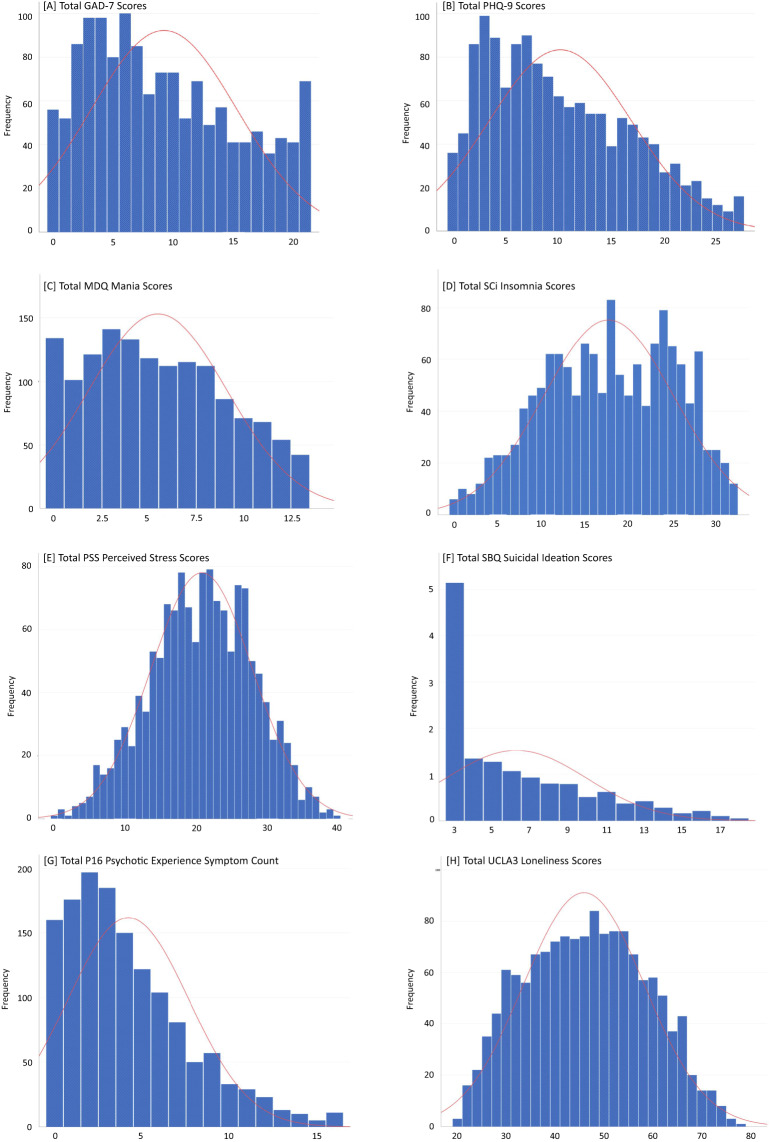
Distribution of individual items for each psychometric measure assessing psychiatric symptoms in the current dataset. (**A**) GAD-7: Anxiety scores ranged from a possible 0–21. (**B**) PHQ-9 Depression scores ranged from a possible 0–27. (**C**) MDQ Mania scores ranged from a possible 0–13. (**D**) SCI Insomnia scores ranged from a possible 0–32. (**E**) PSS Perceived Stress scores ranged from a possible 0–40. (**F**) SBQ-R Suicidal Ideation scores ranged from a possible 3–18. (**G**) P16 Psychotic Like Experiences symptom count ranged from a possible 0–16. (**H**) UCLA3 Loneliness scores ranged from a possible 20–80.

For the current purpose, the SAV file was exported from the Qualtrics online survey platform (Qualtrics, Provo, UT) where the data was cleaned using SPSS v. 29.0 (IBM Corp., Armonk, NT, USA). Descriptive statistics for all calculated variables were inspected to ensure that results fell within am expected range. Whilst normality was assessed using histograms (see Fig. 1), it is vital to consider the inherent skew observed when examining psychiatric prevalence data where a traditional disruption would not be expected and would possibly be of concern. As noted, reliability analysis was performed for each psychometric measure. Here, the internal consistency (Cronbach’s alpha) of each scale remained acceptable, ranging between *α* = 0.822 and 0.936. The dataset was carefully inspected for abnormal response patterns and completion times before any formal analyses was conducted. Neither were observed.

### Prevalence data

The descriptive statistics (means and standard deviations), and Pearson’s bivariate correlations (two-tailed) between each psychiatric symptom are provided in Table 3. In addition, the charts displaying the prevalence of anxiety, depression, mania, insomnia, perceived stress, suicidal ideation, psychotic experiences, and loneliness based upon standardised cut-off points for each respective measure are presented in Table 4.

**Table 3:** Descriptive statistics (means and standard deviations), and correlation matrix for measures of psychiatric symptoms across all participants, ungrouped.

	Mean	SD	Range	1.	*2*.	3.	4.	5.	6.	7.
1. Anxiety	9.25	6.09	0–21	—						
2. Depression	10.16	6.73	0–27	0.775^*^	—					
3. Mania	5.46	3.67	0–13	0.272^*^	0.323^*^	—				
4. Insomnia	17.72	7.47	0–32	−0.542^*^	−0.646^*^	−0.244^*^	—			
5. Perceived Stress	20.88	7.21	0–40	0.739^*^	0.750^*^	0.255^*^	−0.515^*^	—		
6. Suicidal Ideation	6.24	3.70	3–18	0.505^*^	0.632^*^	0.311^*^	−0.415^*^	0.518^*^	—	
7. Psychotic Experiences	4.21	3.46	0–16	0.466^*^	0.551^*^	0.443^*^	−0.370^*^	0.458^*^	0.476^*^	—
8. Loneliness	45.89	12.33	20–77	0.554^*^	0.638^*^	0.267^*^	−0.405^*^	0.606^*^	0.525^*^	0.527^*^

**Note:** SD: Standard Deviation; Anxiety: GAD-7; Depression: PHQ-9; Mania: MDQ; Insomnia: SCI; Perceived Stress: PSS; Suicidal Ideation: SBQ-R; Psychotic Experiences: PQ-16; Loneliness: UCLA3.

*Sig at <0.001

**Table 4:** The prevalence of anxiety, depression, mania, insomnia, perceived stress, suicidal ideation, psychotic experiences and loneliness based upon validated and recommended cut-off points for each respective measure.

Measure	% of Total Sample (N)
**GAD-7: Anxiety**	
Minimal	27.70% (390)
Mild	28.48% (401)
Moderate	21.31% (300)
Severe	22.51% (317)
**PHQ-9: Depression**	
Minimal	25.21% (355)
Mild	27.70% (390)
Moderate	20.31% (286)
Moderately Severe	15.84% (223)
Severe	10.94% (154)
**MDQ: Mania**	
Low-Risk	61.08% (860)
Significant-Risk	38.92% (548)
**SCI: Insomnia**	
Insomnia Not Probable	55.82% (786)
Probable Insomnia	44.18% (622)
PSS: Stress	
Low	15.48% (218)
Moderate	60.94% (858)
Significantly High	23.58% (332)
**SBQ-R: Suicidal Ideation**	
Low-Risk	62.73% (882)
At Risk	37.27% (524)
**PQ-16: Psychotic Experiences**	
Low-Risk	70.41% (990)
At Risk	29.59% (416)
**UCLA3: Loneliness**	
Low	32.81% (462)
Medium	33.10% (466)
High	34.09% (480)

**Note:** See measures section for instructions on data scoring and participant grouping.

### Data limitations

The cross-sectional nature limits the ability to draw any definitive explanation when concerning causal relationships. Next, the sample was not homogeneous, including a disproportionate amount of data from young white female respondents. Finally, potential users of the current data should consider limitations associated with subjective assessments which rely on self-reported information.

### Supplementary information

Supplementary Materials (Appendix A)

## Data Availability

No custom code was used during the compilation of the dataset.
